# Supplementary material for: Predicting treatment response from longitudinal images using multi-task deep learning
Source: Nat Commun. 2021 Mar 25;12:1851. doi: 10.1038/s41467-021-22188-y (PMC7994301; doi:10.1038/s41467-021-22188-y)
Supplement: Supplementary file 1 — Supplementary Information [file 41467_2021_22188_MOESM1_ESM.pdf]

SUPPLEMENTARY INFORMATION

Predicting Treatment Response from Longitudinal Images

Using Multi-Task Deep Learning

Jin et al.

Supplementary Methods

Image acquisition

All patients enrolled in this study underwent multiparametric MRI scans before and after neoadjuvant chemoradiotherapy (CRT). MRI sequences included T1-weighted imaging with and without contrast, T2-weighted imaging, and diffusion-weighted imaging (DWI). Both pre- and post-CRT images for the same patient were obtained using one of two MRI scanners: 1.5T GE Brivo™ MR355, and 3.0 T GE Discovery™ MR750 (GE Healthcare, Milwaukee, WI, USA), which accounted for about one and three quarters of patients in the study, respectively. Gadolinium-based agents were used for acquiring contrast-enhanced T1-weighted MRI. DWI were obtained with two b values: 0 and 1000 sec/mm^2. Details of MRI scanning protocol and acquisition parameters are shown in Table S1.

Image processing

We applied several image processing and harmonization techniques to ensure robust model. First, we corrected for B1 field inhomogeneity effects by using the N4ITK method[1], which is an improved version of N3 (nonparametric nonuniformity normalization) method and publicly available from the Insight Toolkit. To compensate for the effects of different MRI scanning protocols, we applied the ComBat method[2] to harmonize image intensity values across institutions. This was done separately for each MRI sequence, with the institution (internal or external) as the variable of interest, *i.e.*, batch. All images were resampled to a uniform in-plane spatial resolution of 0.5×0.5 mm^2. The original multiparametric MR images were stored at the Sixth Affiliated Hospital of Sun Yat-sen University and image processing was performed using in-house computational platforms.

For each patient, we co-registered the pre- and post-nCRT images in 3D by using rigid registration, given the fixed anatomy of the pelvis. Specifically, we first performed 3D rigid image registration (translation and rotation) between T1-weighted and T2-weighted MRI using normalized mutual information as the loss function. We then registered the diffusion-weighted image to the T2-weighted image with affine transformation in order to correct for the eddy

current distortion and motion effects. Finally, all MRI sequences were aligned to the same spatial coordinates with T2-weighted image as the reference. Image registration was implemented using the extensively benchmarked elastix software in ITK[3]. The registered images were visually checked for quality assurance by the radiologist. Deformable registration is not appropriate for our purpose because the goal is to capture changes in the tumor between the two images. Uncertainty in registration was taken into account during network training (see below).

For network training and validation purposes, the primary tumor contour was manually delineated in all slices on the pre- and post-CRT MR images by two radiologists in consensus. The gross tumor and invasive disease in surrounding tissue were included in the tumor contour. Regional lymph nodes were not contoured. In the post-treatment images, abnormal anatomic structures including residual tumor and any treatment-induced changes were contoured. Both radiologists were blinded to the pathologic outcome when contouring. Any discrepancy in tumor contours was resolved by a third radiologist with 15 years of experience in the interpretation of body imaging. All tumor contours were reviewed, and their quality was checked by a biomedical engineer with 8 years of experience in image processing. As a preprocessing procedure, a synthetic speed image was created from the original multi-parametric images by a clustering algorithm in the ITK-SNAP software (www.itksnap.org). The speed image has values in the range -1 to 1, and the goal is to make voxels inside the tumor having positive speed values, and non-tumor voxels having negative speed values. It is worth noting that the purpose of the speed image is to help generate a manual delineation of the tumor. It is not used or needed for the prediction of deep learning model. Based on this initial segmentation, a radiologist then reviewed and fine-tuned the tumor region to generate a more precise tumor delineation. This allowed the user to take into consideration both anatomical and functional characteristics across different imaging contrasts in the multiparametric MRI.

We focused on a volume of interest with a size of 256×256×16 that centered around the primary tumor. This volume also included satellite foci and nearby tissues such as blood vessels or

lymph nodes with potential tumor invasion. All four images (T1w, T1w+C, T2w, DWI) were used. Finally, a tensor image with a size of 256×256×16×4 was fed into the network for training.

Network architecture

The proposed multi-task learning network architecture consists of a Siamese subnetwork for feature extraction and tumor segmentation, and a response prediction subnetwork (Figure S1).

<u>*Siamese segmentation sub-network*</u> The Siamese sub-network contains two networks of the same structure that share identical parameters for the segmentation task. Here we adopt a modified 3D U-Net architecture, which includes a contracting path, an expansive path, and skip connections. At each level in the contracting path, a $3 \times 3 \times 3$ convolution with stride 2 is performed to double the number of feature maps while reducing the resolution. This is followed by a context module or pre-activation residual block with two $3 \times 3 \times 3$ convolutional layers and a dropout layer in between. Each level of the expansive path begins with a localization module consisting of a $3 \times 3 \times 3$ convolution, which up-samples the feature maps to a higher resolution. A direct concatenation is used to combine the up-sampled features with the features at the corresponding level in the contracting path. Finally, different levels of the expansive path are combined via elementwise summation, which produces the final segmentation result.

<u>*Response prediction sub-network*</u> This sub-network is used to predict the status of pCR from pre/post-therapy MRI images. The premise is that the well-trained Siamese segmentation sub-network can capture salient features from the tumor target area and even spreading to the whole image, which will benefit response prediction. Here, we combine the extracted features from three different abstraction levels: (1) intermediate layer in the contracting path, (2) intermediate layer of the elementwise summation combination module at the end of the network and (3) the bottom layer of the U-shaped network. By doing so, we ensure that both shallow and deep feature representations obtained by the Siamese segmentation sub-network can be fully integrated and applied to the response prediction task.

Rather than simply concatenate the features of the pre- and post-therapy images, we performed depth-wise convolution between the corresponding feature maps. This was implemented by treating one feature map as the convolution kernel and performing convolution operation on each corresponding channel along the depth axis. Since the two feature maps have the same size, the output is a one-dimensional vector as calculated below:

$$Y_Z = \sum_{0 \le x \le w, 0 \le y \le h} X^{(1)}_{x,y,z} \cdot X^{(2)}_{x,y,z} \qquad z = 1 \dots d \tag{1}$$

where $X^{(1)}_{x,y,z}$ and $X^{(2)}_{x,y,z}$ denote feature maps extracted from pre and post-therapy images, respectively. Y_z denotes zth value of the output vector and w, h, d are the width, height, depth of the feature map.

The results of depth-wise convolution from the three different layers are combined via concatenation. The final prediction output is obtained after the activation layer using the sigmoid function.

Loss function

The loss function of the multi-task learning network consists of two parts. The first part is the loss used for segmentation. We are not only concerned about the overall morphological changes of the primary tumor, but also the tumor invasion into surrounding vasculature and lymph nodes. Using traditional volume-based Dice loss alone cannot measure these small but critical changes at the tumor periphery. Therefore, we combined the Dice score and fractal dimension[4] as the total loss for segmentation:

$$L_{seg} = \frac{2\sum_i u_i v_i}{\sum_i u_i + \sum_i v_i} + |FD_u - FD_v| \tag{2}$$

where u_i and v_i denote the softmax output of segmentation sub-network and ground truth at voxel i; FD_u and FD_v denote the fractal dimension of the segmentation result and ground truth. Here, we used a variant of the box counting method to calculate the fractal dimension of the tumor contour[5].

The second part is loss for binary classification in the response prediction sub-network. Here,

we used the focal loss[6] to address the class-imbalance issue in our dataset.

$$L_{class} = -\alpha(1 - p_t)^{\gamma}\log(p_t) \tag{3}$$

where y is ground-truth class and p denotes the model output. p_t equals p when y equals 1 and equals $1 - p$ otherwise. We set α to be 0.25 and γ to be 2.

The final loss function is a weighted linear combination of the two parts:

$$Loss = w_{class}L_{class} + \sum_{i=1}^{2} w_{seg,i}L_{seg,i} \tag{4}$$

The optimal weights were determined by cross validation. Here, we set w_{class} as 1 and both w_{seg} as 0.2 so that more attention is focused on response prediction.

Network training

We employed several established techniques to minimize the risk of over-fitting, including cross validation, data augmentation, instance normalization, early stopping, and learning rate decay during the training process. We used cross validation to tune the hyper-parameters including the weight for loss function, learning rate, batch size, patience for early stopping, and decay factor.[10] In this study, the batch size was set to 32. The learning rate was set to 0.01 and learning rate decay was 0.5. We used instance normalization instead of traditional batch normalization since the latter may be destabilized by small batch sizes[7].

Data augmentation is widely used for training deep neural networks to increase the number and diversity of training samples and has been shown to improve model generalizability. Importantly, data augmentation was designed with the goal to handle image intensity variations in different MRI protocols as well as to simulate shifts to account for registration uncertainty. Specifically, the data augmentation included image rotation with an angle randomly sampled from (-10°, +10°), random shifts with 0-10% of the total image width and height, image smoothing and enhancement with Gaussian filtering and Laplacian filtering, and adding noise. Here, we applied data augmentation on the fly when generating training batches by using the ImageDataGenerator toolbox available in the Keras software platform.

Radiomics model for pCR prediction

Radiomics features were extracted from both pre-CRT and post-CRT multiparametric MRI using the PyRadiomics software package[8]. For each MRI sequence, a total of 1074 3D radiomics features were extracted: 1) 271 first-order features reflecting the distribution of voxel intensities within tumor region; 3) 803 texture features: including computational features based on gray level co-occurrence matrix (n=351): gray level run length matrix (n=226), and gray level size zone matrix (n=226). These features quantitatively describe the higher-order voxel-level heterogeneity in the tumor. In addition, 21 3D shape features describing the tumor morphological and structural features were calculated. In training set (321 cases of MRIs after nCRT), the optimal subset of radiomics features associated with the response of nCRT (pCR and non-pCR) was selected by applying the minimum-redundancy-maximum-relevance technique. Finally, we built a logistic regression model combined with the least absolute shrinkage and selection operator (LASSO) to predict the response and tested the performance in the validation cohorts.

Supplementary Figures

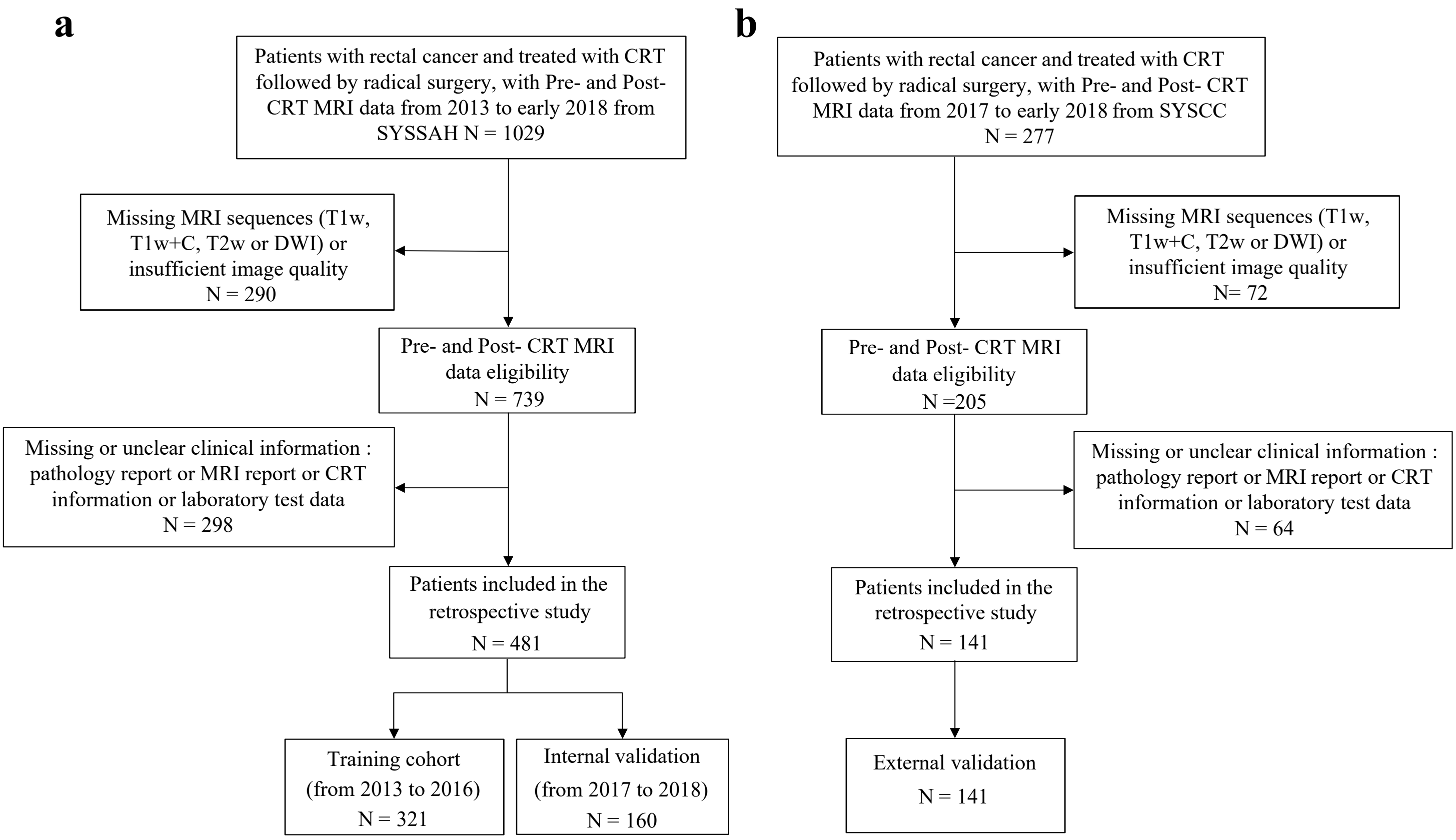

Supplementary Fig. 1. Flowchart shows inclusion and exclusion criteria from SYSSAH (a) and SYSCC (b). SYSSAH: Sun Yat-sen University, the Sixth Affiliated Hospital; SYSCC: Sun Yat-sen University Cancer Center.

a

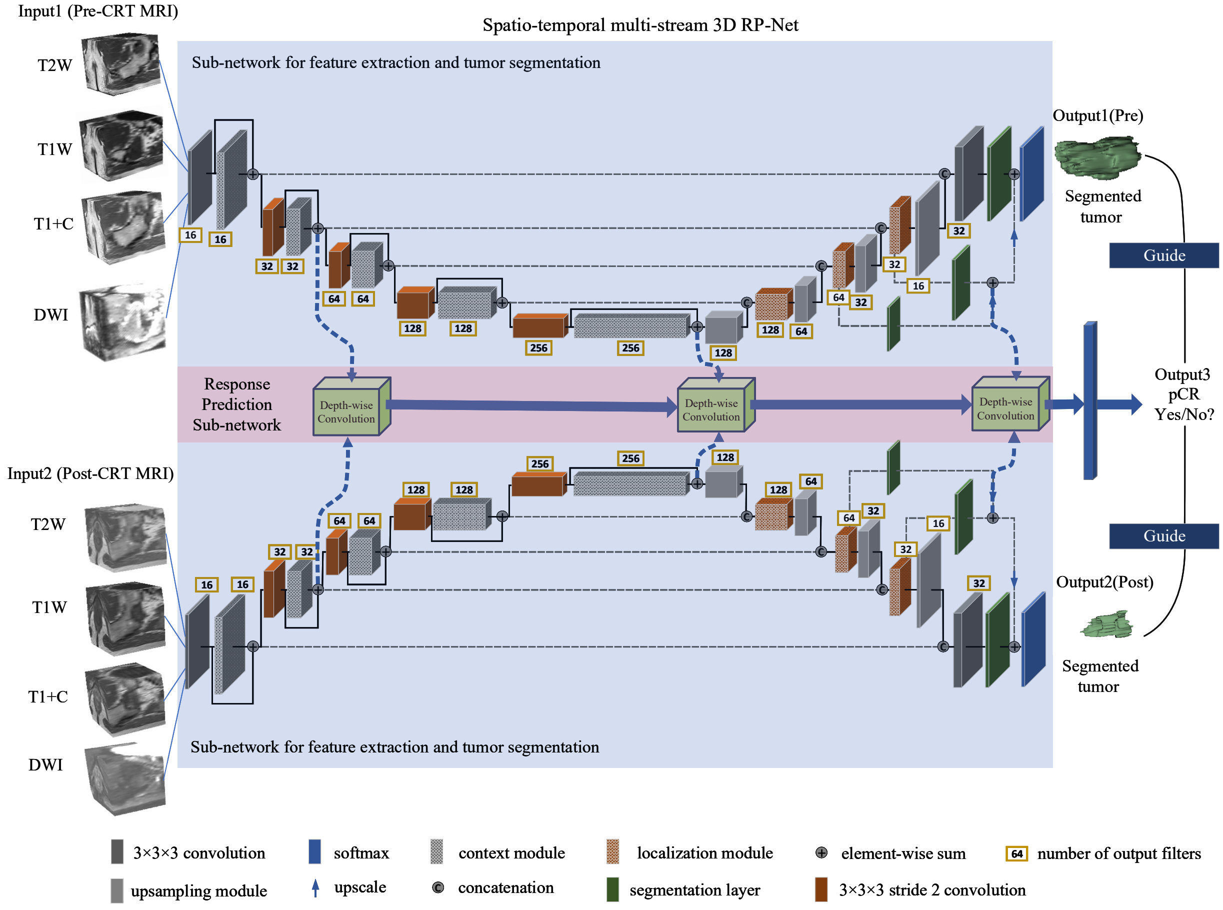

b

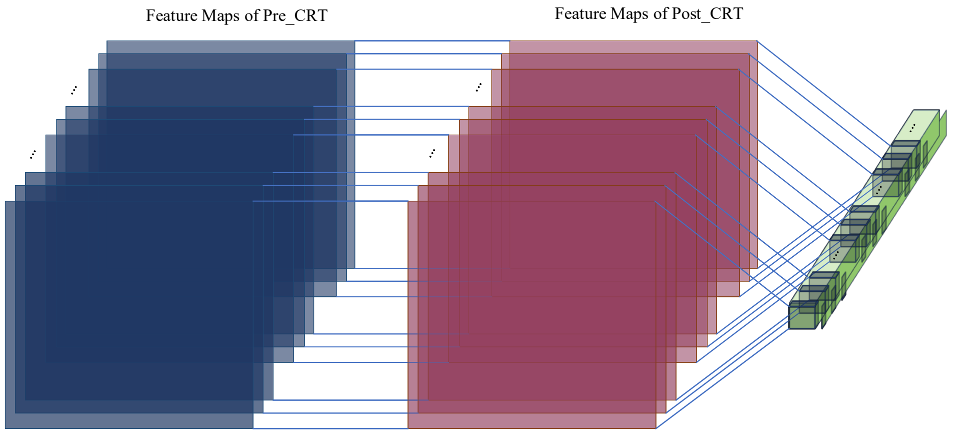

Supplementary Fig. 2. Details of the proposed 3D RP-Net architecture. a. The network consists of two subnetworks: one for feature extraction and tumor segmentation, and one for response prediction. **b.** Depth-wise convolution in the response prediction subnetwork.

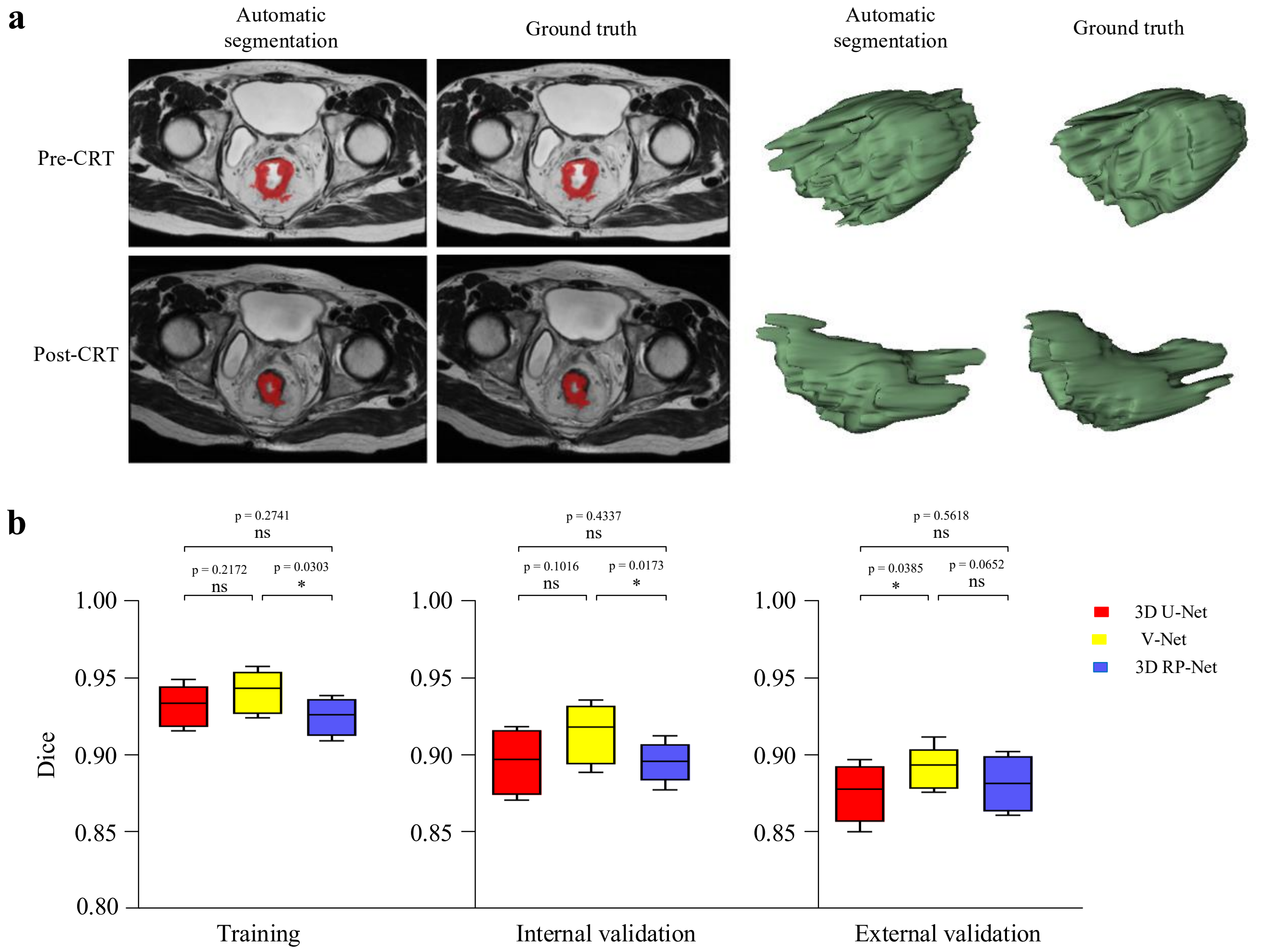

Supplementary Fig. 3. Tumor segmentation results. a. Pre- and post-CRT images of a patient with non-pCR. From left to right: segmentation result and ground truth at an image slice, 3D rendering for the segmentation result and ground truth. **b.** Comparison of Dice in the training (n = 321 patients), internal validation (n = 160 patients) and external validation (n = 141 patients) sets by three segmentation methods 3D U-Net, V-Net and 3D RP-Net. The Dice coefficient between expert contours was 0.92 ± 0.03 (mean, standard deviation) for all tumors. In the box plots, the central line represents the median, the bounds of box correspond to the first and third quartiles, and the whiskers are the minimum and maximum of the data. P values were computed based on the two-side t test between the Dice coefficients for different models and adjusted for multiple comparisons. ns, not significant; * : $0.01 \leq P < 0.05$.

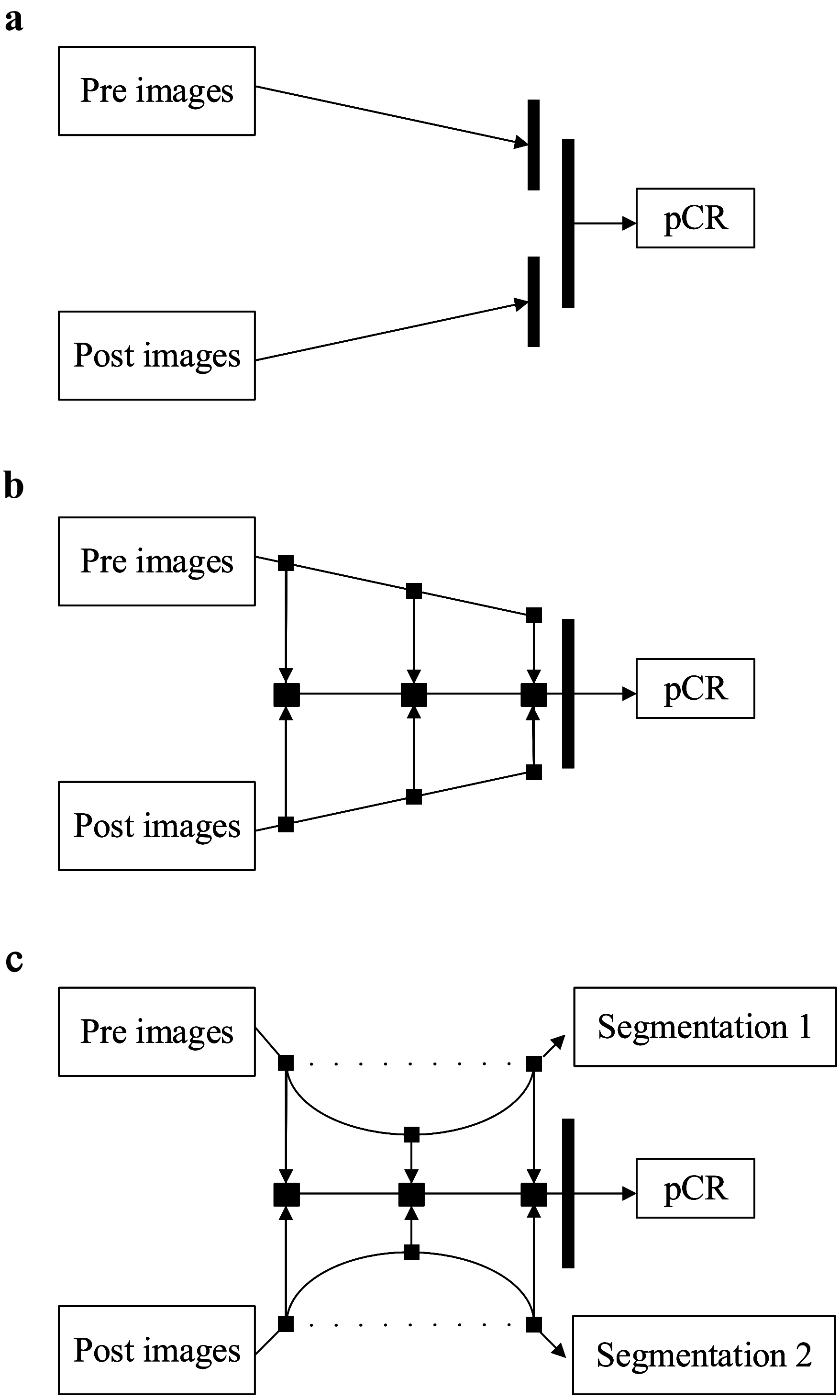

Supplementary Fig. 4. Schematic design of three network architectures that use longitudinal images for response prediction. a. Traditional Siamese network: simple concatenation at the fully connected layer, single-task learning. **b.** Improved Siamese network: multi-scale feature integration, single-task learning. **c**. Proposed network: multi-scale feature integration, multi-task learning (tumor segmentation and response prediction).

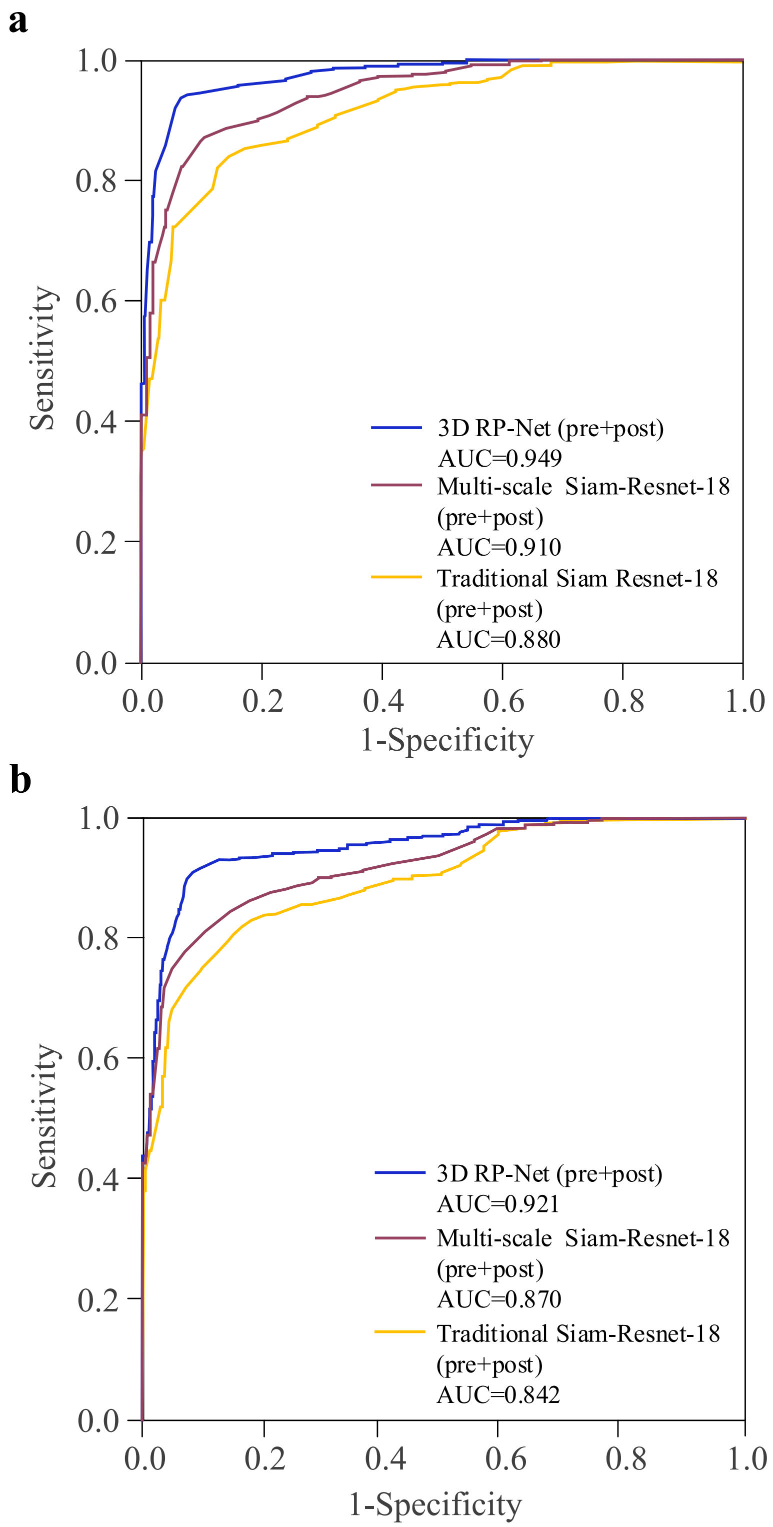

Supplementary Fig. 5. ROC curves for response prediction of three network models shown in Fig. S3 in the internal validation (a) and external validation (b) cohorts.

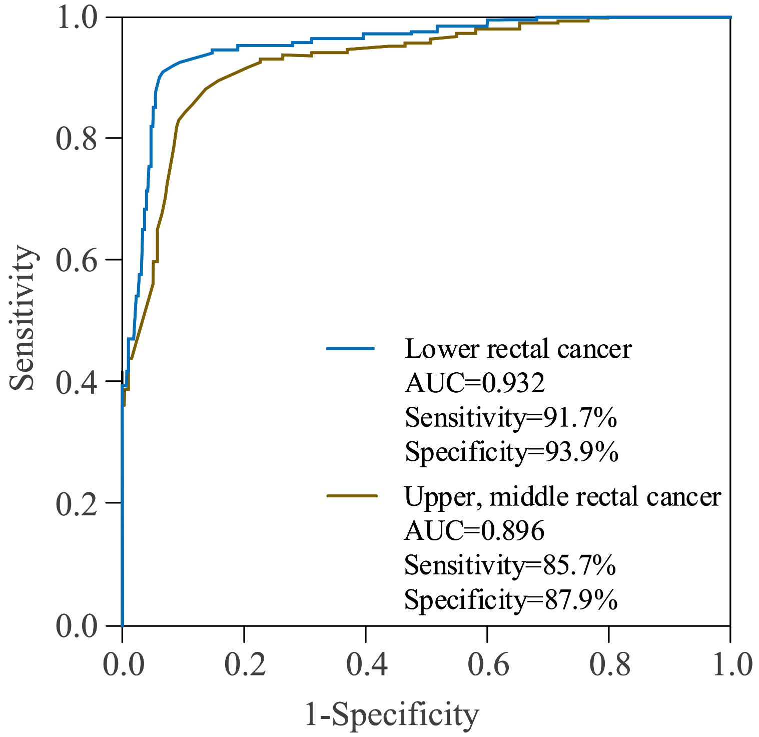

Supplementary Fig. 6. ROC curves for response prediction of the proposed network model in the subgroup of patients with upper, middle and lower rectal cancer in the external validation cohort.

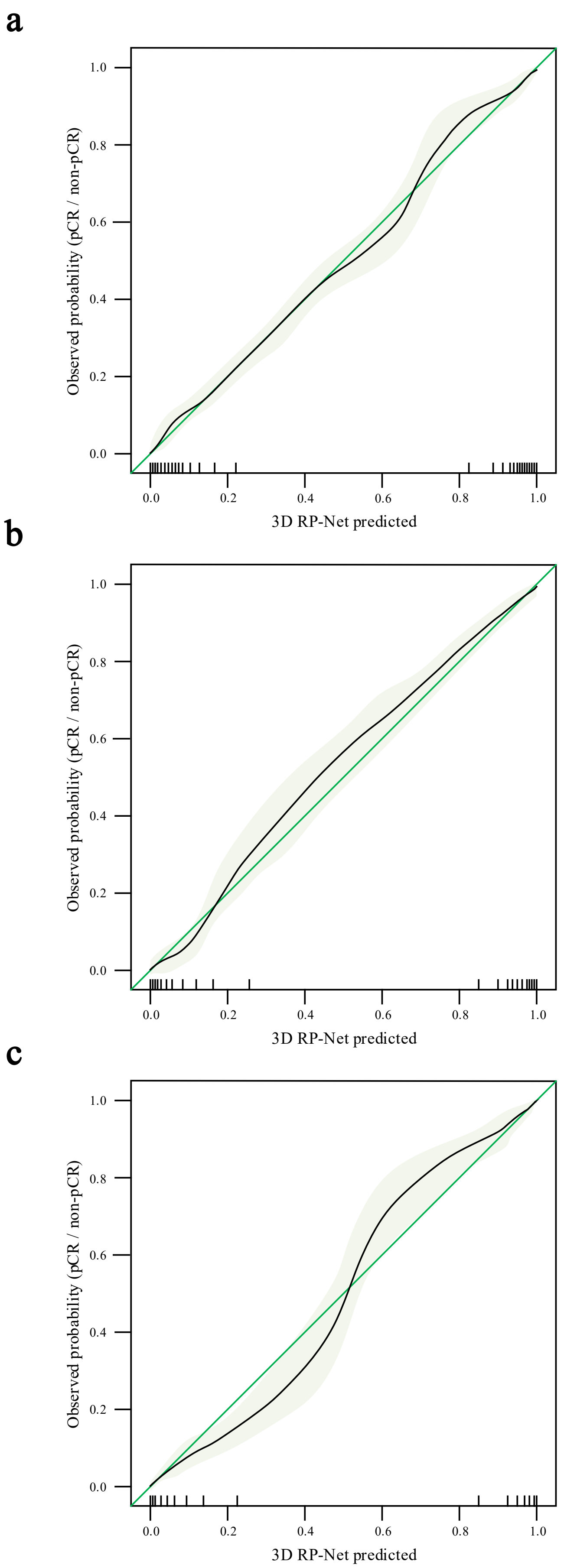

Supplementary Fig. 7. Calibration curve for response prediction of the proposed network model in the training (a), internal validation (b), and external validation (c) cohorts. The solid line corresponds to the mean calibration curve. The light-colored band around the calibration curve represents the 95% confidence level. The vertical bar on the X-axis represents the distribution of predicted scores for each patient. represents the distribution of predicted scores for each patient.

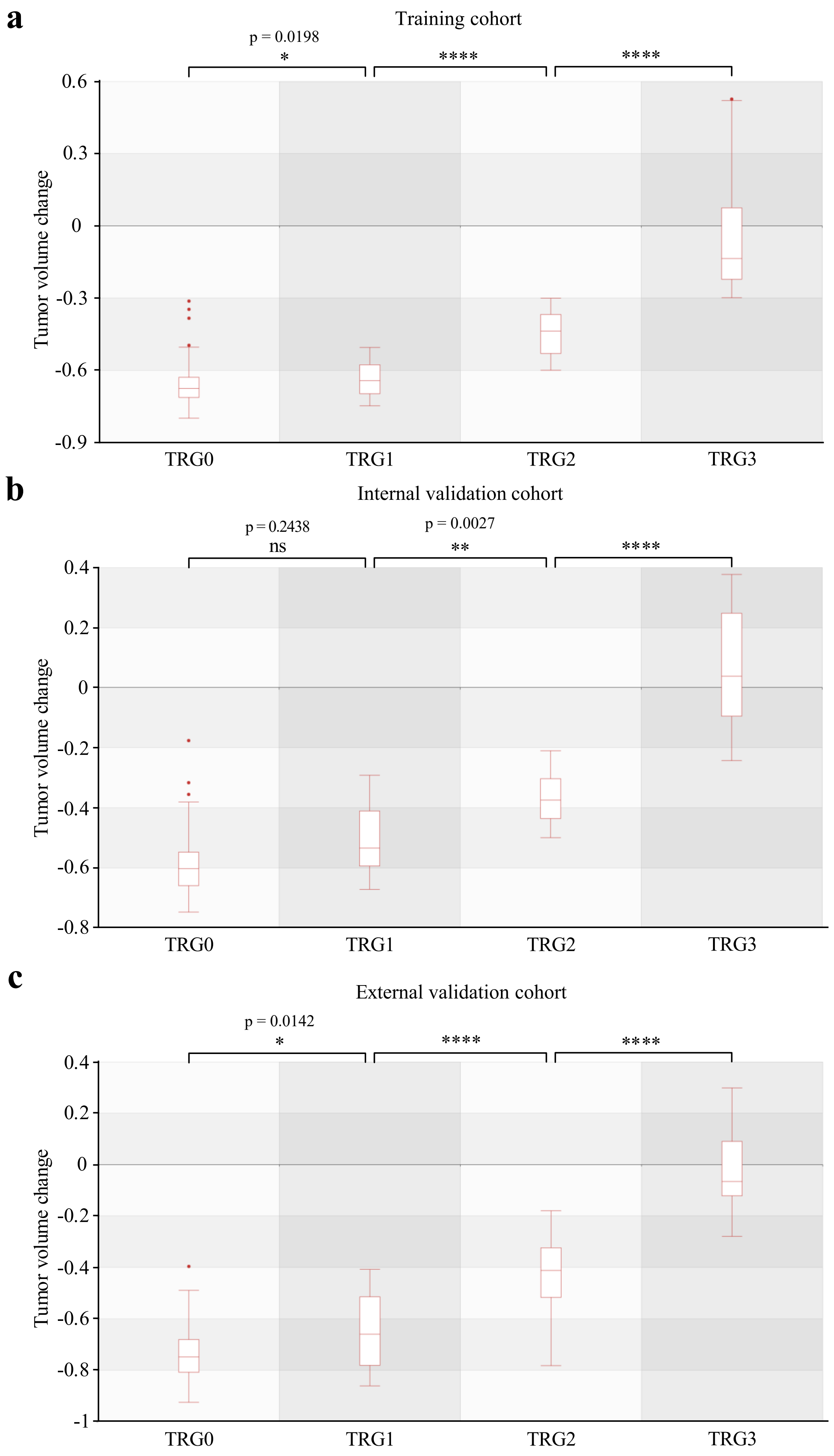

Supplementary Fig. 8. Tumor volume change of neoadjuvant chemotherapy (nCRT) for the four tumor regression grades (TRG) in the training (a), internal validation (b), and external validation (c) cohorts. Tumor volume change = (Post-Volume – Pe-Volume)/Pre-Volume. In the box plots, the central line represents the median, the bounds of box correspond to the first and third quartiles, and the whiskers represent the minimum and maximum (if no outliers) or 1.5X interquartile range (outliers shown as dots). P values were computed based on the two-side t test between consecutive TRG groups and adjusted for multiple comparisons. The number of patients in the TRG0, TRG1, TRG2, TRG3 groups were: n = 60, 85, 129, 47 in the training cohort; n = 45, 26, 60, 29 in the internal validation cohort; n = 43, 37, 47, 14 in the external validation cohort, respectively. ns, not significant; * : 0.01 ≤ P < 0.05; ** : 0.001 ≤ P < 0.01; *** : 0.0001 ≤ P < 0.001; **** : P < 0.0001.

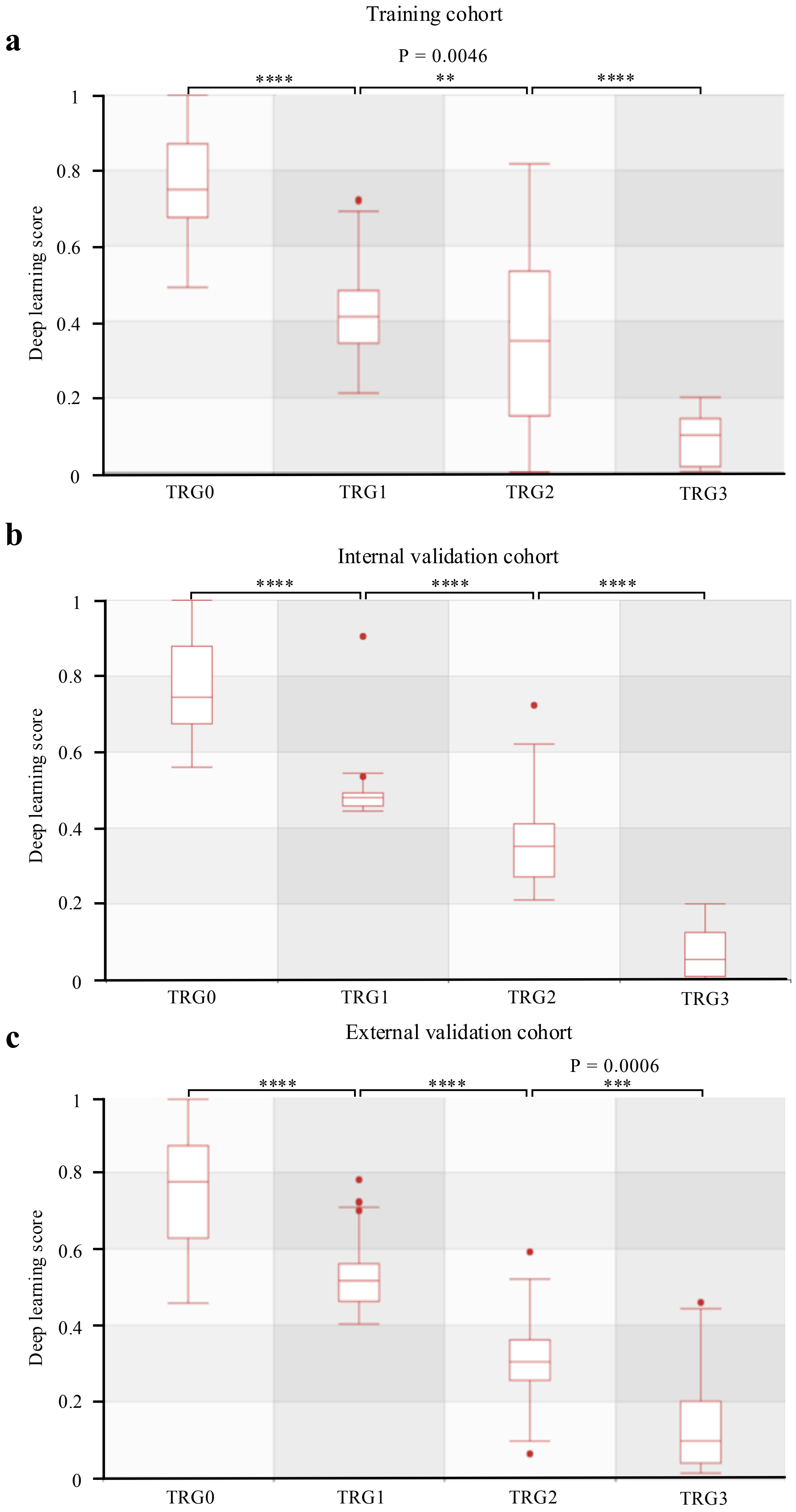

Supplementary Fig. 9. Distribution of deep learning scores predicted by 3D RP -Net model for the four tumor regression grades in the training (a), internal validation (b), and external validation (c) cohorts. TRG: tumor regression grade. TRG0 corresponds to pCR; TRG1-3 correspond to non-pCR. In the box plots, the central line represents the median, the bounds of box correspond to the first and third quartiles, and the whiskers represent the minimum and maximum (if no outliers) or 1.5X interquartile range (outliers shown as dots). P values were computed based on the two-side t test between consecutive TRG groups and adjusted for multiple comparisons. The number of patients in the TRG0, TRG1, TRG2, TRG3 groups were: n = 60, 85, 129, 47 in the training cohort; n = 45, 26, 60, 29 in the internal validation cohort; n = 43, 37, 47, 14 in the external validation cohort, respectively. * : $0.01 \leq P < 0.05$; ** : $0.001 \leq P < 0.01$; *** : $0.0001 \leq P < 0.001$; **** : $P < 0.0001$.

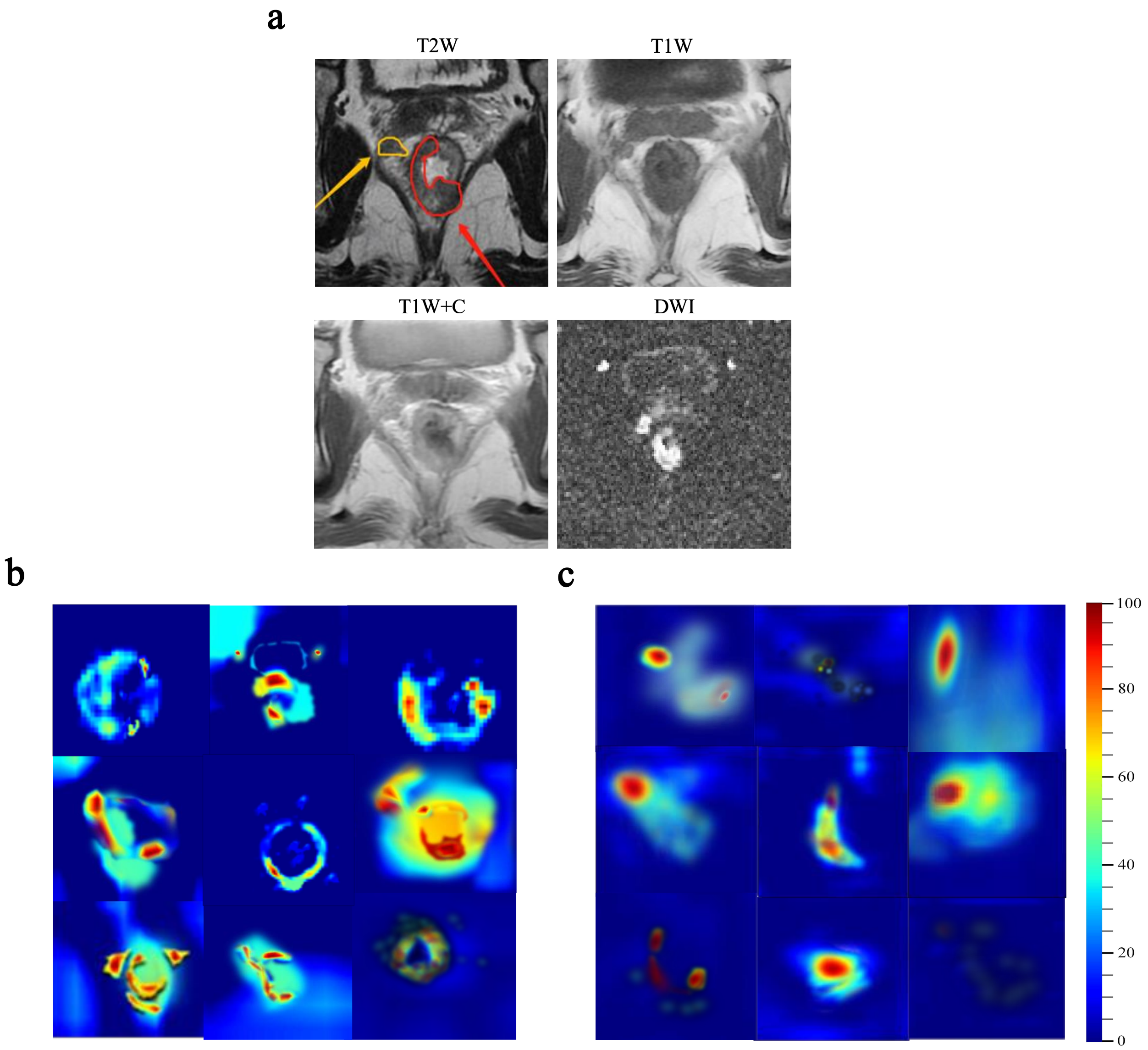

Supplementary Fig. 10. MRI and corresponding feature maps generated in the response prediction subnetwork of 3D RP-Net. a. Four MRI sequences (T1W, T1W+C, T2W, DWI). **b.** Feature maps at shallow layers, which mainly reflect structural information on high-resolution T1W and T2W MRI, such as tumor boundary, shape, and texture. **c.** Features maps at deep layers, which mainly represent high-level semantic tumor characteristics from anatomical images as well as functional information contained in DWI. Only one representative case in the external validation cohort was shown here. Overall, this pattern was observed in most patients.

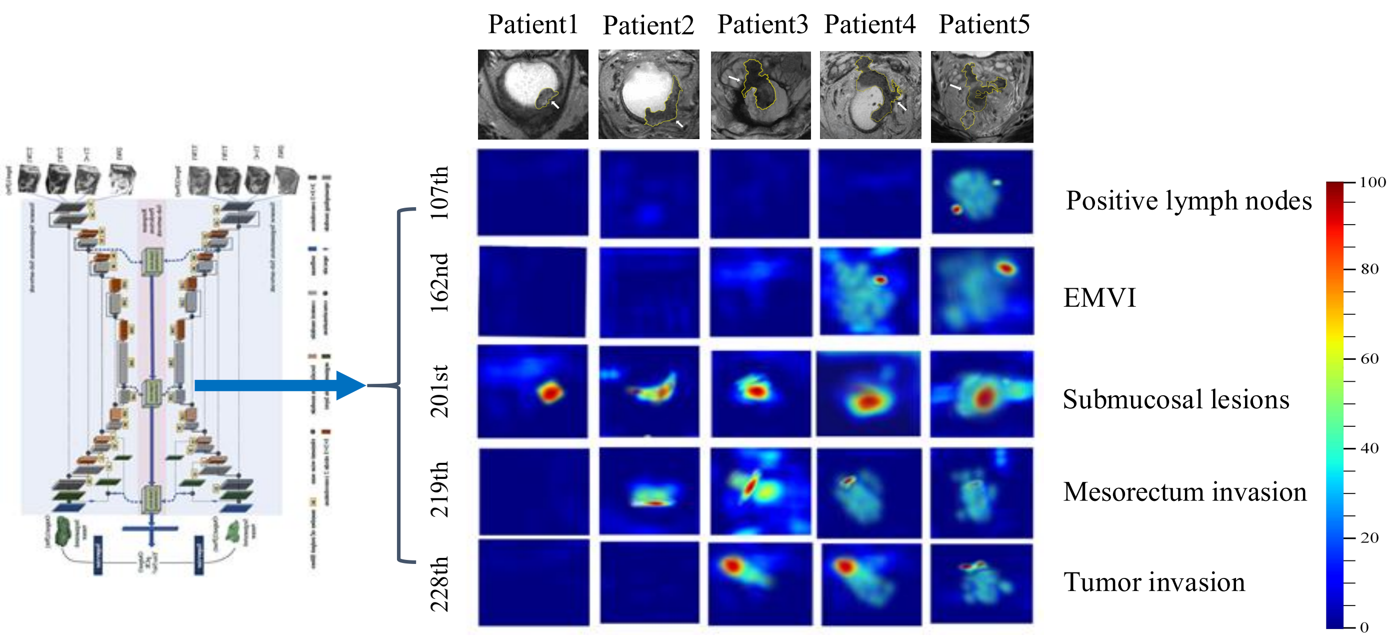

Supplementary Fig. 11. Visualization for representative channels at the intermediate layer in the response prediction subnetwork of 3D RP-Net. Only five representative patients are shown here. Overall, the positive lymph node pattern was activated in n=214,128,110 patients; the EMVI pattern was activated in n=137,86,65 patients; the submucosal lesion pattern was activated in n=248,147,130 patients; the mesorectum invasion pattern was activated in 289,141,121 patients; tumor invasion was activated in 29, 13, 8 patients, in the training, internal validation, and external validation cohort, specifically. Due to depth-wise convolution, only a small number of channels are activated in each layer. EMVI: extramural vascular invasion. Mesorectum invasion: Tumor invasion into the perirectal fat that surrounds the rectum.

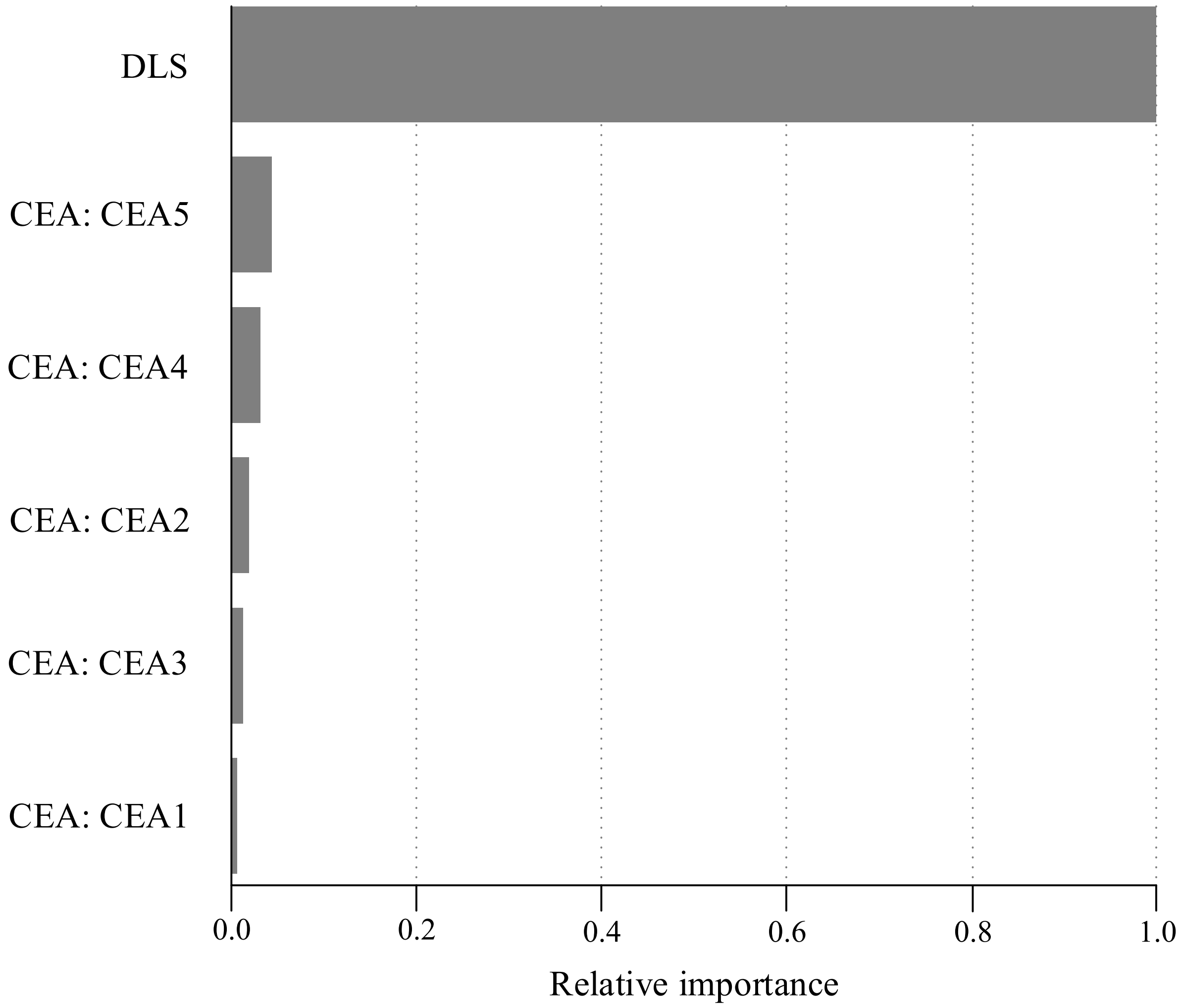

Supplementary Fig. 12. Relative importance of the predictor variables in the random forest model when combining imaging and CEA data. DLS, deep learning score. CEA: carcinoembryonic antigen. The variables CEA1-5 correspond to five discrete categories based on the clearance patterns given pre/post-therapy CEA level as defined in **Fig. 5a**.

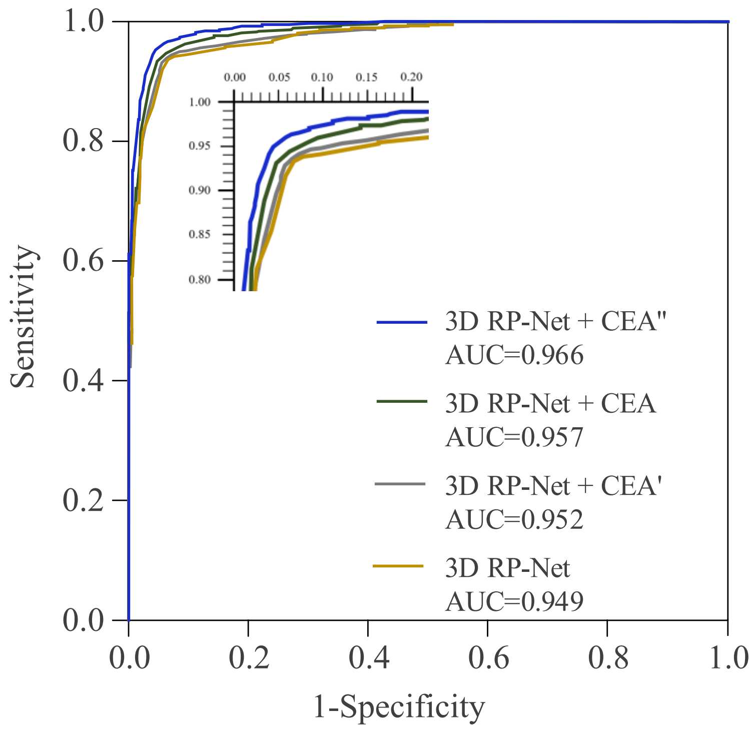

Supplementary Fig. 13. ROC curves for response prediction of imaging integrated with different CEA models in the internal validation cohort. CEA model uses the binary category given post-therapy CEA level: CEA <5ug/L and CEA ≥5ug/L; CEA' model uses the continuous value of post-therapy CEA; CEA" model uses five discrete categories based on the clearance patterns given pre/post-therapy CEA level as defined in **Fig. 5a**. The difference in AUC between the deep learning model and 3 different integrated models was not statistically significant (DeLong's test $p > 0.05$, adjusted for multiple comparisons).

Data Access Agreement

The Sixth Affiliated Hospital (SAH) of Sun Yat-sen University and the Recipient Institution (RECIPIENT) hereby enter into this Agreement for the transfer of data used in the paper by Jin C, et al. Predicting treatment response from longitudinal images using multi-task deep learning.

In consideration of SAH providing data to RECIPIENT, RECIPIENT hereby agrees to the following terms and conditions:

1. Data will be provided to RECIPIENT with a Research Plan that is approved by SAH.

2. Data will be used only by RECIPIENT for purpose described in the Research Plan.

3. The RECIPIENT will not release data to a third party without prior approval from SAH.

4. The RECIPIENT will not share, publish, or otherwise release any findings or conclusions derived from analysis of data obtained from SAH without prior approval from SAH.

5. All data transferred to RECIPIENT shall remain the property of SAH.

____

For the Sixth Affiliated Hospital of Sun Yat-sen University

Date: ______________________________

____

For the RECIPIENT

Date: ______________________________

Supplementary Figure 14. A copy of the Data Access Agreement

Supplementary Table 1. MRI scanning protocol and acquisition parameters.

	Training and internal validation cohorts				External validation cohort			
**Sequence**	**DWI**	**T1W+C**	**T1W**	**T2W**	**DWI**	**T1W+C**	**T1W**	**T2W**
**TR (ms)**	2750-5650	340-610		2720-5200	4200-7200	530-750		4800-6900
**TE (ms)**	85 or 97	13		120-130	60-70	7.5-9.0		72-93
**ETL**	1	3		19	1	3		28 or 21
**Pixel Bandwidth (Hz/pixel)**	1953.12	122.07		122.07	1953.12	325.508		488.28
**Pixel Spacing**	1.5625, 1.5625	0.4883, 0.4883		0.5469, 0.5469	1.4844, 1.4844	0.7422, 0.7422		0.4297, 0.4297
**Acquisition Matrix**	192*192	320*224		320*224	128*128	384*320		384*384 or 320*320
**Slice Thickness(mm)**	5	5		5	5	5		3 or 5
**Magnetic field strength**	1.5T of 67.4 %, 3.0T of 32.6%				3.0T of 81.3% , 1.5T of 18.7%			

Note: TR, Repetition Time; TE, Echo Time; ETL, Echo Train Length.

Supplementary Table 2. The performance of different deep learning models in the internal validation cohort.

Methods	Accuracy (95% CI)	AUROC (95% CI)	Sensitivity (95% CI)	Specificity (95% CI)	PPV (95% CI)	NPV (95% CI)
**Resnet-18 (Pre)**	83.13% (76.51%-88.19%)	0.811 (0.738-0.872)	75.00% (59.35%-86.30%)	86.21% (78.27%-91.67%)	67.35% (52.34%-79.64%)	90.09% (82.58%-94.71%)
**Resnet-18 (Post)**	81.88% (75.14%-87.12%)	0.829 (0.760-0.898)	72.73% (56.96%-84.54%)	85.34% (77.29%-90.99%)	65.31% (50.29%-77.94%)	89.19% (81.52%-94.04%)
**Siam-Resnet-18 (Pre+Post)**	86.25% (79.99%-90.81%)	0.880 (0.823-0.927)	84.09% (69.33%-92.84%)	87.06% (79.30%-92.34%)	71.15% (56.73%-82.45%)	93.52% (86.64%-97.13%)
**3D RP-Net (Pre+Post)**	**93.75%** (88.75%-96.70%)	**0.949** (0.913-0.979)	**93.18%** (80.29%-98.22%)	**93.96%** (87.52%-97.33%)	**85.42%** (71.62%-93.45%)	**97.32%** (91.79%-99.30%)

Note: PPV, positive predictive value; NPV, negative predictive value.

Supplementary Table 3. Performance of different deep learning models in the external validation cohort.

Methods	Accuracy (95% CI)	AUROC (95% CI)	Sensitivity (95% CI)	Specificity (95% CI)	PPV (95% CI)	NPV (95% CI)
**Resnet-18 (Pre)**	79.43% (71.98%-85.33%)	0.760 (0.676-0.829)	72.09% (56.09%-84.17%)	82.65% (73.40%-89.29%)	64.58% (49.40%-77.45%)	87.10% (78.16%-92.87%)
**Resnet-18 (Post)**	78.01% (70.45%-84.10%)	0.797 (0.718-0.876)	69.77% (53.70%-82.33%)	81.63% (72.26%-88.47%)	62.50% (47.33%-75.68%)	86.02% (76.92%-92.05%)
**Siam-Resnet-18 (Pre+Post)**	83.69% (76.65%-88.94%)	0.842 (0.771-0.901)	81.40% (66.08%-91.08%)	84.69% (75.69%-90.90%)	70.00% (55.22%-81.71%)	91.21% (82.93%-95.85%)
**3D RP-Net (Pre+Post)**	**91.49%** (85.59%-95.19%)	**0.921** (0.868-0.963)	**90.70%** (76.95%-96.98%)	**91.84%** (84.08%-96.15%)	**82.98%** (68.65%-91.86%)	**95.74%** (88.85%-98.63%)

Supplementary Table 4. Detailed information for prediction performance of the proposed model using only T2WI in the study cohorts.

Cohort	Accuracy (95% CI)	AUROC (95% CI)	Sensitivity (95% CI)	Specificity (95% CI)	PPV (95% CI)	NPV (95% CI)
**Training**	88.47% (84.49%-91.55%)	0.944 (0.915-0.972)	84.21% (71.63%-92.09%)	89.39% (84.88%-92.72%)	63.16% (51.26%-73.71%)	96.33% (92.91%-98.20%)
**Internal validation**	83.75% (77.20%-88.72%)	0.884 (0.831-0.937)	79.55% (64.25%-89.67%)	85.34% (77.29%-90.99%)	67.31% (52.78%-79.28%)	91.67% (84.35%-95.88%)
**External validation**	**80.85%** (73.52%-86.54%)	**0.857** (0.792-0.923)	**76.74%** (61.00%-87.72%)	**82.65%** (73.40%-89.29%)	**66.00%** (51.14%-78.41%)	**89.01%** (80.29%-94.32%)

Note: PPV, positive predictive value; NPV, negative predictive value.

Supplementary Table 5. Detailed information for prediction performance of the radiomics model in the study cohorts.

Cohort	Accuracy (95% CI)	AUROC (95% CI)	Sensitivity (95% CI)	Specificity (95% CI)	PPV (95% CI)	NPV (95% CI)
**Training**	90.97% (87.29%-93.67%)	0.931 (0.901-0.962)	89.47% (77.81%-95.65%)	91.29% (87.05%-94.28%)	68.92% (56.96%-78.89%)	97.57% (94.53%-99.01%)
**Internal validation**	83.75% (77.20%-88.72%)	0.889 (0.838-0.940)	84.09% (69.33%-92.84%)	83.62% (75.35%-89.61%)	66.07% (52.09%-77.84%)	93.27% (86.15%-97.02%)
**External validation**	**80.85%** (73.52%-86.54%)	**0.860** (0.796-0.924)	**79.07%** (63.52%-89.42%)	**81.63%** (72.26%-88.47%)	**65.38%** (50.84%-77.67%)	**89.89%** (81.21%-94.98%)

Note: PPV, positive predictive value; NPV, negative predictive value.

Calculation formula for the radiomics model: 3.05325 + 3.91318 * $OSS_{Post\text{-}DWI}$ + 2.85370 * $OGIV_{Pre\text{-}T2W}$ + 0.55842 * $LS\text{-}3DGSZNUN_{Post\text{-}T2W}$ + 1.02681 * $WLHL\text{-}GC_{Post\text{-}T1W}$ + 1.40150 * $OGDNUN_{Post\text{-}DWI}$ + 2.32681 * $LS3DGI_{Post\text{-}T1W+C}$ + 1.91541* $OGI_{Pre\text{-}T1}$ + 4.08659 * $WLLL\text{-}GDA_{Post\text{-}T2W}$ + 1.31185 * $OFRMS_{Pre\text{-}DWI}$ + 1.90421 * $WHHLGGLNU_{Post\text{-}TIW}$ + 1.03187 * $WLLH\text{-}GGLV_{Post\text{-}T2W}$ + 3.28516 * $WHHH\text{-}GDE_{Post\text{-}T2}$ + 0.78931 * $WHLH\text{-}GJE_{Post\text{-}T1W}$ + 0.94217 * $WLLH\text{-}GI_{Post\text{-}T1W+C}$ - 0.56831 * $LS\text{-}3DGC_{Pre\text{-}T1W}$ + 1.78126 * $WLHLGZE_{Post\text{-}T2W}$ + 0.70585 * $LS\text{-}3D\text{-}GDV_{Pre\text{-}T1W}$ + 1.31129 * $LS\text{-}3DGSE_{Post\text{-}T2W}$ + 2.61341 * $WHLH\text{-}GLDLGLE_{Post\text{-}T1W}$ + 1.09199 * $LS\text{-}3DGJE_{Pre\text{-}T2W}$ + 1.11420 * $LS\text{-}3DGDE_{Post\text{-}T1W.}$

Abbreviations: original_glcm_InverseVariance (OGIV); original_shape_Sphericity (OSS); log-sigma-2-0-mm-3D_glszm_SizeZoneNonUniformityNormalized (LS-3DGSZNUN); wavelet-LHL_glcm_Correlation (WLHL-GC); original_gldm_DependenceNonUniformityNormalized (OGDNUN); log-sigma-2-0-mm-3D_glcm_Id (LS3DGI); original_glcm_Idmn (OGI); wavelet-LLL_glcm_DifferenceAverage (WLLL-GDA); original_firstorder_RootMeanSquared (OFRMS); wavelet-HHL_glszm_GrayLevelNonUniformity (WHHLGGLNU); wavelet-LLH_glszm_GrayLevelVariance (WLLH-GGLV); wavelet-HHH_gldm_DependenceEntropy (WHHH-GDE); wavelet-HLH_glcm_JointEnergy (WHLH-GJE); wavelet-LLH_glcm_Imc2 (WLLH-GI); log-sigma-5-0-mm-3D_glcm_Contrast (LS-3DGC); wavelet-LHL_glszm_ZoneEntropy (WLHLGZE); log-sigma-1-0-mm-3D_gldm_DependenceVariance (LS-3D-GDV); log-sigma-2-0-mm-3D_glcm_SumEntropy (LS-3DGSE); wavelet-HLH_gldm_LargeDependenceLowGrayLevelEmphasis(WHLH-GLDLGLE); log-sigma-3-0-mm-3D_glcm_JointEnergy (LS-3DGJE); log-sigma-1-0-mm-3D_gldm_DependenceEntropy (LS-3DGDE).

Supplementary Table 6. Performance of 3D RP-Net in the subset of upper, middle and lower rectal cancer in the internal validation cohort.

Tumor location	Accuracy (95% CI)	AUROC (95% CI)	Sensitivity (95% CI)	Specificity (95% CI)	PPV (95% CI)	NPV (95% CI)
**Upper, middle rectal cancer**	90.16% (79.81%-95.75%)	0.925 (0.851-0.985)	88.24% (62.25%-97.94%)	90.91% (77.42%-97.05%)	78.95% (53.90%-93.03%)	95.24% (82.58%-99.17%)
**Lower rectal cancer**	93.94% (87.15%-97.45%)	0.948 (0.898-0.989)	92.59% (74.25%-98.71%)	94.44% (85.65%-98.21%)	86.21% (67.43%-95.49%)	97.14% (89.14%-99.50%)

Note: We divided the cohort into two subsets: upper, middle rectal cancer with 17 pCR, 44 non-pCR in 61 patients, prevalence 27.87% (17.51% - 41.03%) and lower rectal cancer with 27 pCR, 72 non-pCR in 99 patients, prevalence 27.27% (19.03%-37.30%).

Supplementary Table 7. Performance of 3D RP-Net in the subset of upper, middle and lower rectal cancer in the external validation cohort.

Location	Accuracy (95% CI)	AUROC (95% CI)	Sensitivity (95% CI)	Specificity (95% CI)	PPV (95% CI)	NPV (95% CI)
**Upper, middle rectal cancer**	87.34% (78.05%-93.17%)	0.896 (0.815-0.966)	85.71% (62.64%-96.24%)	87.93% (76.09%-94.61%)	72.00% (50.40%-87.13%)	94.44% (83.66%-98.55%)
**Lower rectal cancer**	93.33% (81.48%-98.37%)	0.932 (0.843-0.987)	91.67% (59.74%-99.56%)	93.94% (78.38%-98.94%)	84.62% (53.66%-97.29%)	96.88% (82.00%-99.84%)

Note: We divided the cohort into two subsets: upper, middle rectal cancer with 21 pCR, 58 non-pCR in 79 patients, prevalence 26.58% (17.56% - 37.91%) and lower rectal cancer with 12 pCR, 33 non-pCR in 45 patients, prevalence 26.67% (15.10%-42.21%).

Supplementary Table 8. Performance of 3D RP-Net by gender in the internal validation cohort.

**Male**	94.96% (89.21%-97.90%)	0.962 (0.922-0.997)	94.12% (78.94%-98.97%)	95.29% (87.73%-98.48%)	88.89% (73.00%-96.38%)	97.59% (90.76%-99.58%)
**Female**	92.68% (79.88%-98.17%)	0.952 (0.882-0.993)	90.00% (54.12%-99.48%)	93.55% (77.16%-98.87%)	81.82% (47.76%-96.79%)	96.67% (80.95%-99.83%)

Note: We divided the cohort into two subsets: Male rectal cancer with 34 pCR, 85 non-pCR in 119 patients, prevalence 28.57% (20.85% - 37.70%) and Female rectal cancer with 10 pCR, 31 non-pCR in 41 patients, prevalence 24.39% (12.91%-40.64%).

Supplementary Table 9. Performance of 3D RP-Net by gender in the external validation cohort.

Gender	Accuracy (95% CI)	AUROC (95% CI)	Sensitivity (95% CI)	Specificity (95% CI)	PPV (95% CI)	NPV (95% CI)
**Male**	92.78% (85.61%-96.70%)	0.938 (0.879-0.997)	92.31% (73.40%-98.66%)	92.96% (83.65%-97.38%)	82.76% (63.51%-93.47%)	97.06% (88.84%-99.49%)
**Female**	90.91% (78.29%-96.96%)	0.904 (0.802-0.993)	88.24% (62.25%-97.94%)	92.59% (74.25%-98.71%)	88.24% (62.25%-97.94%)	92.59% (74.25%-98.71%)

Note: We divided the cohort into two subsets: Male rectal cancer with 26 pCR, 71 non-pCR in 97 patients, prevalence 26.80% (18.55% - 36.92%) and female rectal cancer with 17 pCR, 27 non-pCR in 44 patients, prevalence 38.64% (24.75%-54.49%).

Supplementary Table 10. Performance of 3D RP-Net in the subset of magnetic field strength in the internal validation cohort.

Magnetic field strength	Accuracy (95% CI)	AUROC (95% CI)	Sensitivity (95% CI)	Specificity (95% CI)	PPV (95% CI)	NPV (95% CI)
**1.5T**	94.44% (88.16%-97.67%)	0.950 (0.923-0.983)	93.33% (76.49%-98.84%)	94.87% (86.69%-98.34%)	87.50% (70.07%-95.92%)	97.37% (89.95%-99.54%)
**3.0T**	96.15% (86.28%-99.68%)	0.969 (0.921-0.992)	92.86% (64.17%-99.63%)	97.37% (84.57%-99.86%)	92.86% (64.17%-99.63%)	97.37% (84.57%-99.86%)

Note: We divided the cohort into two subsets: the magnetic field strength of 1.5T with 30 pCR, 78 non-pCR in 108 patients, prevalence 27.78% (19.80% - 37.36%) and magnetic field strength of 3.0T with 14 pCR, 38 non-pCR in 52 patients, prevalence 26.92% (16.00%-41.27%).

Supplementary Table 11. Performance of 3D RP-Net in the subset of magnetic field strength in the external validation cohort.

Magnetic field strength	Accuracy (95% CI)	AUROC (95% CI)	Sensitivity (95% CI)	Specificity (95% CI)	PPV (95% CI)	NPV (95% CI)
**1.5T**	88.00% (69.21%-96.67%)	0.919 (0.803-0.997)	87.50% (46.68%-99.34%)	88.24% (62.25%-97.94%)	77.78% (40.19%-96.05%)	93.75% (67.71%-99.67%)
**3.0T**	93.97% (87.86%-97.26%)	0.959 (0.917-0.996)	91.43% (75.81%-97.76%)	95.06% (87.16%-98.41%)	88.89% (73.00%-96.38%)	96.25% (88.68%-99.3%)

Note: We divided the cohort into two subsets: the magnetic field strength of 1.5T with 8 pCR, 17 non-pCR in 25 patients, prevalence 32.00% (15.73% - 53.55%) and magnetic field strength of 3.0T with 35 pCR, 81 non-pCR in 116 patients, prevalence 30.17% (22.18%-39.50%).

Supplementary Table 12. Performance of 3D RP-Net and integrated models with different CEA variables in the internal validation cohort.

Methods	Accuracy (95% CI)	AUROC (95% CI)	Sensitivity (95% CI)	Specificity (95% CI)	PPV (95% CI)	NPV (95% CI)
**3D RP-Net**	93.75% (88.75%-96.70%)	0.949 (0.906-0.980)	93.18% (80.29%-98.22%)	93.96% (87.52%-97.33%)	85.42% (71.62%-93.45%)	97.32% (91.79%-99.30%)
**3D RP-Net +CEA**	95.00% (90.29%-97.60%)	0.957 (0.918-0.986)	93.18% (80.29%-98.22%)	95.69% (89.73%-98.40%)	89.13% (71.62%-93.45%)	97.36% (91.79%-99.30%)
**3D RP-Net + CEA'**	94.38% (89.52%-97.15%)	0.952 (0.912-0.985)	93.18% (80.29%-98.22%)	94.83% (88.62%-97.88%)	87.23% (73.56%-94.70%)	97.34% (91.86%-99.31%)
**3D RP-Net +CEA"**	**95.63%** (91.08%-98.03%)	**0.966** (0.931-0.993)	**95.45%** (83.30%-99.21%)	**95.69%** (89.74%-98.40%)	**89.36%** (76.11%-96.02%)	**98.23%** (93.12%-99.69%)

Note: CEA model uses the binary category given post-therapy CEA level: CEA <5ug/L and CEA ≥5ug/L; CEA' model uses the continuous value of post-therapy CEA; CEA" model uses five discrete categories based on the clearance patterns given pre/post-therapy CEA level as defined in Fig. 5a.

Supplementary Reference

1. Tustison, N.J., *et al.* N4ITK: Improved N3 Bias Correction. *Ieee T Med Imaging* **29**, 1310-1320 (2010).
2. Orlhac, F., Frouin, F., Nioche, C., Ayache, N. & Buvat, I. Validation of a Method to Compensate Multicenter Effects Affecting CT Radiomics. *Radiology* **291**, 52-58 (2019).
3. Klein, S., Staring, M., Murphy, K., Viergever, M.A. & Pluim, J.P. elastix: a toolbox for intensity-based medical image registration. *IEEE Trans Med Imaging* **29**, 196-205 (2010).
4. Baumann, G., Barth, A. & Nonnenmacher, T.F. Measuring fractal dimensions of cell contours: practical approaches and their limitations. in *Fractals in biology and medicine* 182-189 (Springer, 1994).
5. Piantanelli, A., *et al.* Fractal characterisation of boundary irregularity in skin pigmented lesions. *Medical and Biological Engineering and Computing* **43**, 436-442 (2005).
6. Lin, T.-Y., Goyal, P., Girshick, R., He, K. & Dollár, P. Focal loss for dense object detection. in *Proceedings of the IEEE international conference on computer vision* 2980-2988 (2017).
7. Ulyanov, D., Vedaldi, A. & Lempitsky, V.J.a.p.a. Instance normalization: The missing ingredient for fast stylization. *arXiv preprint* (2016).
8. van Griethuysen, J.J.M., *et al.* Computational Radiomics System to Decode the Radiographic Phenotype. *Cancer Res* **77**, e104-e107 (2017).
