# Supplementary material for: Predicting treatment response from longitudinal images using multi-task deep learning
Source: Nat Commun. 2021 Mar 25;12:1851. doi: 10.1038/s41467-021-22188-y (PMC7994301; doi:10.1038/s41467-021-22188-y)
Supplement: Supplementary file 3 — Reporting Summary [file 41467_2021_22188_MOESM3_ESM.pdf]

nature research

Corresponding author(s): Ruijiang Li

Last updated by author(s): Feb 20, 2021

Reporting Summary

Nature Research wishes to improve the reproducibility of the work that we publish. This form provides structure for consistency and transparency in reporting. For further information on Nature Research policies, see our Editorial Policies and the Editorial Policy Checklist.

Statistics

For all statistical analyses, confirm that the following items are present in the figure legend, table legend, main text, or Methods section.

n/a	Confirmed	
☐	☒	The exact sample size (n) for each experimental group/condition, given as a discrete number and unit of measurement
☐	☒	A statement on whether measurements were taken from distinct samples or whether the same sample was measured repeatedly
☐	☒	The statistical test(s) used AND whether they are one- or two-sided<br>*Only common tests should be described solely by name; describe more complex techniques in the Methods section.*
☐	☒	A description of all covariates tested
☐	☒	A description of any assumptions or corrections, such as tests of normality and adjustment for multiple comparisons
☐	☒	A full description of the statistical parameters including central tendency (e.g. means) or other basic estimates (e.g. regression coefficient) AND variation (e.g. standard deviation) or associated estimates of uncertainty (e.g. confidence intervals)
☐	☒	For null hypothesis testing, the test statistic (e.g. F, t, r) with confidence intervals, effect sizes, degrees of freedom and P value noted<br>*Give P values as exact values whenever suitable.*
☒	☐	For Bayesian analysis, information on the choice of priors and Markov chain Monte Carlo settings
☒	☐	For hierarchical and complex designs, identification of the appropriate level for tests and full reporting of outcomes
☐	☒	Estimates of effect sizes (e.g. Cohen's d, Pearson's r), indicating how they were calculated

Our web collection on statistics for biologists contains articles on many of the points above.

Software and code

Policy information about availability of computer code

Data collection	All data used in this study are preexisting data in standard formats; no special software was used to collect the data.
Data analysis	The deep learning models were developed using standard libraries in open-source platforms including Keras (version 2.2) and TensorFlow (version 1.14). Custom codes for the deployment of the model are available https://github.com/Heng14/3D_RP-Net. Tumor delineation was performed using the open-source software ITK-SNAP (version 3.8.0). Radiomic features were computed using the open-source software PyRadiomics (version 3.0).

For manuscripts utilizing custom algorithms or software that are central to the research but not yet described in published literature, software must be made available to editors and reviewers. We strongly encourage code deposition in a community repository (e.g. GitHub). See the Nature Research guidelines for submitting code & software for further information.

Data

Policy information about availability of data

All manuscripts must include a data availability statement. This statement should provide the following information, where applicable:

- Accession codes, unique identifiers, or web links for publicly available datasets
- A list of figures that have associated raw data
- A description of any restrictions on data availability

Source data that were used to generate the figures and tables in this study are provided as separate data files. The clinical and MRI data are not publicly available for patient privacy protection purposes but are available from the corresponding authors (X.W. and F.G.) with a signed data access agreement.

Field-specific reporting

Please select the one below that is the best fit for your research. If you are not sure, read the appropriate sections before making your selection.

- [x] Life sciences
- [ ] Behavioural & social sciences
- [ ] Ecological, evolutionary & environmental sciences

For a reference copy of the document with all sections, see nature.com/documents/nr-reporting-summary-flat.pdf

Life sciences study design

All studies must disclose on these points even when the disclosure is negative.

Sample size	With approval from the institutional review board, we retrospectively collected data for a total of 622 patients diagnosed with locally advanced rectal cancer. A formal power calculation was not performed in this retrospective study. Instead, sample size was determined empirically by collecting the largest number of samples possible given the pre-specified inclusion/exclusion criteria described in the Methods. For training and validation purposes, we recruited all eligible patients who were treated with neoadjuvant chemoradiotherapy followed by total mesorectal excision during a recent 5-year period and for whom both pre/post-treatment MRI scans and pathology report were available at the time of the study. The detailed flowchart for patient enrollment is shown in Figure S1.
Data exclusions	The data exclusion criteria are described in the Methods and Supplementary Information. In general, patients with insufficient MRI image quality, incomplete images before and after treatment, missing or unclear pathology information were excluded.
Replication	We assessed the reproducibility of the deep learning model by independently testing its performance for predicting pathologic complete response in internal validation and external validation cohorts.
Randomization	This is not relevant for our study as there was no experimental group.
Blinding	The clinical and pathology data were blinded to the investigator during tumor contouring. The pathologic response data were available to the the investigator during model development, but were blinded to the model develper for internal and external validation of the model.

Reporting for specific materials, systems and methods

We require information from authors about some types of materials, experimental systems and methods used in many studies. Here, indicate whether each material, system or method listed is relevant to your study. If you are not sure if a list item applies to your research, read the appropriate section before selecting a response.

Materials & experimental systems

n/a	Involved in the study	
☒	☐	Antibodies
☒	☐	Eukaryotic cell lines
☒	☐	Palaeontology and archaeology
☒	☐	Animals and other organisms
☐	☒	Human research participants
☒	☐	Clinical data
☒	☐	Dual use research of concern

Methods

n/a	Involved in the study	
☒	☐	ChIP-seq
☒	☐	Flow cytometry
☒	☐	MRI-based neuroimaging

Human research participants

Policy information about studies involving human research participants

Population characteristics	This information is presented for each of the study cohorts in Table 1.
Recruitment	The recruitment criteria for the data in this study are listed in the Methods and supplementary information. Specifically, we recruited all eligible patients who met the following criteria: (1) pathologically confirmed diagnosis of locally advanced rectal adenocarcinoma; (2) treatment with neoadjuvant chemoradiotherapy (CRT) followed by total mesorectal excision; (3) both pre-CRT and post-CRT MRI scans (T1w with and without contrast, T2w, DWI) available within one week prior to the initiation of CRT and surgery.<br>Patients who had other concurrent malignancies or had previously received anticancer treatment were excluded. Some patients were also excluded due to technical issues such as missing image or pathology information or poor image quality. The patients enrolled in the study had a broad range of characteristics with both genders and across various age groups included (Table 1).
Ethics oversight	The IRB of the participating centers (The Sixth Affiliated Hospital of Sun Yat-sen University, Guangzhou, China; Sun Yat-sen Cancer Center, Guangzhou, China) approved the study.

Note that full information on the approval of the study protocol must also be provided in the manuscript.
